# Supplementary material for: Analysis of PPARγ Signaling Activity in Psoriasis
Source: Int J Mol Sci. 2021 Aug 10;22(16):8603. doi: 10.3390/ijms22168603 (PMC8395241; doi:10.3390/ijms22168603)
Supplement: Supplementary file 1 [file ijms-22-08603-s001.zip › Supplemental materials_Analysis of PPARg signaling activity in psoriasis/Pathway models/Models images and html files/PPARG expressed in cells assotiated with psoriasis/225527.html]

dendritic cell


# CellType dendritic cell

|  |  |
| --- | --- |
| URN | urn:agi-ncimcelltype:C0011306 |
| Total Entities | 81 |
| Connectivity | 8078 |
| Name | dendritic cell |
| ObjectType | Cell |

---

|  |  |
| --- | --- |
| ChildConcepts | dendritic cell differentiation |
|  | dendritic cell migration |
|  | dendritic cell chemotaxis |
|  | dendritic cell apoptosis |
|  | conventional dendritic cell |
|  | plasmacytoid dendritic cell |
|  | CD8+ dendritic cell |
|  | CD8- dendritic cell |
|  | CD4+ dendritic cell |
|  | activated dendritic cell |
|  | veiled cell |
|  | bone marrow derived dendritic cell |
|  | follicular dendritic cell |
|  | interdigitating dendritic cell |
|  | interdigitating reticulum cell |
|  | dendritic cell development |
|  | dendritic cell transendothelial migration |
|  | dendritic cell function |
|  | dendritic cell infiltration |
|  | dendritic cell structure |
|  | dendritic cell survival |
|  | dendritic cell phenotype |
|  | dendritic cell population |
|  | dendritic cell branching |
|  | dendritic cell recognition |
|  | dendritic cell adhesion |
|  | dendritic cell formation |
|  | dendritic cell motion |
|  | dendritic cell proliferation |
|  | dendritic cell homing |
|  | dendritic cell sequestration |
|  | dendritic cell repertoire |
|  | dendritic cell interaction |
|  | dendritic cell homeostasis |
|  | dendritic cell motility |
|  | dendritic cell renewal |
|  | dendritic cell localization |
|  | dendritic cell phagocytosis |
|  | dendritic cell cytotoxicity |
|  | dendritic cell fate commitment |
|  | dendritic cell size |
|  | dendritic cell polarity |
|  | dendritic cell contact |
|  | dendritic cell fate |
|  | dendritic cell viability |
|  | dendritic cell disruption |
|  | dendritic cell priming |
|  | dendritic cell invasion |
|  | dendritic cell death |
|  | dendritic cell autophagy |
|  | dendritic cell growth |
|  | dendritic cell developmental process |
|  | dendritic cell lifespan |
|  | dendritic cell aggregation |
|  | dendritic cell architecture |
|  | dendritic cell damage |
|  | dendritic cell extravasation |
|  | dendritic cell rolling |
|  | dendritic cell division |
|  | dendritic cell tethering |
|  | dendritic cell mediated cytotoxicity |
|  | dendritic cell spine density |
|  | dendritic cell distribution |
|  | dendritic cell ratio |
|  | dendritic cell communication |
|  | dendritic cell phagocyte activity |
|  | dendritic cell spreading |
|  | dendritic cell transdifferentiation |
|  | dendritic cell clonal anergy |
|  | dendritic cell fate specification |
|  | dendritic cell macrophage differentiation |
|  | dendritic cell volume |
|  | dendritic cell stress |
|  | dendritic cell compartmentalization |
|  | dendritic cell morphogenesis |
|  | dendritic cell diapedesis |
|  | dendritic cell destruction |
|  | dendritic cell swelling |
|  | dendritic cell transepithelial migration |
|  | dendritic cell senescence |
|  | dendritic cell fate determination |

---

|  |  |
| --- | --- |
| Pathway | IL13/STAT6/ZEB1 Signalling Promotes Cancer Metastatic Progression |
|  | pathway 1 |
|  | PPARG expressed in cells assotiated with psoriasis |
|  | Neighbors of dendritic cell |
|  | Vg9Vd2 cells-\|SARS |
|  | 1\_Intestine epithelial cell dysfunction in ulcerative colitis |
|  | 1\_HIV entrance to the host cell and viral reproduction |
|  | 2\_Defective tolerance of autoreactive T-cell in T1D |

---

|  |  |
| --- | --- |
| MedScan ID | 10000122 |

---

|  |  |
| --- | --- |
| Alias | dendritic cell |
|  | dendritic cells |

---

|  |  |
| --- | --- |
| NCIm ID | C0011306 |

---
